# Interference-Aware Radio Resource Management for Cognitive High-Throughput Satellite Systems

**DOI:** 10.3390/s20010197

**Published:** 2019-12-29

**Authors:** Rui Zhang, Yuhan Ruan, Yongzhao Li, Can Liu

**Affiliations:** State Key Laboratory of Integrated Service Network, Xidian University, Xi’an 710071, China; rui_zhang_xd@163.com (R.Z.); yzhli@xidian.edu.cn (Y.L.); hzjcliucan@163.com (C.L.)

**Keywords:** HTS system, cognitive radio, full frequency reuse, co-channel interference, radio resource management

## Abstract

By providing interactive broadband services to geographical areas underserved by terrestrial infrastructure, multi-beam satellite systems play a central role in future wireless communications. Targeting the terabit throughput requirements in satellite communications, we introduce a cognitive radio-based high-throughput satellite (HTS) system architecture where full frequency reuse is employed among beams. Moreover, by analyzing the characteristics of the considered architecture, we discuss the design challenges of radio resource management in cognitive HTS systems exposed to both intra-system and inter-system co-channel interference. Furthermore, to combat interference with low overhead, we propose a generic interference-aware resource management framework based on joint spatial division and multiplexing (JSDM). Under this framework, user grouping along with two-stage precoding is studied to achieve substantial improvement in the overall system throughput. Finally, some future research directions and challenges are also given.

## 1. Introduction

A multi-faceted fifth-generation (5G) system is expected to have the ability of ubiquitous access to high-data-rate services/applications from any device anytime and anywhere, driving the revolution to a highly efficient network architecture. In this context, satellite communications, which are complementary to terrestrial networks, are becoming an inherent part of 5G and beyond 5G eco-systems [1,2,3]. As a matter of fact, satellite communications can not only provide coverage for areas without backbone connectivity (e.g., maritime, aeronautic, rural, and emergency), but also deliver high capacity broadband services for high-density population scenarios to decongest existing terrestrial wireless networks.

With the ever-increasing demand for high-speed multimedia and broadband services, high-throughput satellite (HTS) systems [4] are emerging as a promising paradigm to provide on-demand coverage and large-capacity communications for maritime networks [5] and Unmanned Aerial Vehicle UAV surveillance [6]. Equipped with an array-fed reflector, the HTS system can produce a large number of beams, enabling interactive broadband services on top of broadcasting. Although a number of Ka-band HTS systems have already been deployed, due to the spectrum shortage, there is still a large gap with respect to the throughput requirement of the next generation of terabit/s satellites within the 2020 horizon [7]. The main reasons and the related solutions are summarized in Table 1.

One reason for the limited throughput is the inefficient utilization of the deployed spectrum in HTS systems. To increase the bandwidth utilization efficiency, the authors in [8] proposed to dynamically allocate the orthogonal bandwidth based on different traffic demands. For an orthogonal frequency division multiple-access-based satellite forward link, the authors in [9] combined the multicast resource allocation schemes and application-layer joint coding to enhance the performance of mobile satellite systems. In [10,11], although the authors proposed dynamic frequency allocation schemes to enhance the frequency flexibility, the conventional four-color frequency reuse (4FR) scheme was still employed to ensure interference isolation at the expense of lower spectrum utilization, which results in a capacity reduction problem. Moreover, the authors in [12] proposed a priority code scheme (PCS) to mitigate the unsuitable allocations of frequency resources in the HTS system, where the frequency-reuse pattern is assumed to be three to provide spatial separation among beams operating on the same frequency. In this way, no interference or little interference is exposed on adjacent beams while the spectrum is underutilized. To boost the achievable spectral efficiency of the broadband high-throughput multibeam satellite systems, a promising approach is to use a more aggressive frequency reuse pattern combined with interference mitigation techniques [13]. In this context, full frequency reuse (FFR) among beams along with multiuser interference detection and cancelation on the user side or an advanced radio resource management (RRM) strategy at the transmitter side is emerging as a promising way towards the efficient utilization of the available spectrum and the increase of the overall system throughput [27]. Specifically, the authors in [14] designed a multiuser detection technique to alleviate the inter-beam interference and investigated the capacity performance of multibeam satellite communication systems using a rain fading model. In [15,16], precoding-based transmission schemes were designed to enable full frequency reuse in multibeam satellite systems.

Another reason for the limited throughput is the exclusive regulation of spectrum assignment. According to ITU-R [17], only a 500 MHz spectrum in the Ka band is exclusively available for HTS, which has been shown to be insufficient to meet the forthcoming demands for broadband satellite services. To alleviate the spectrum scarcity in future satellite communications, employing cognitive radio (CR) technology in satellite communications has emerged as a promising candidate, which is referred as a so-called cognitive satellite–terrestrial network [18,19]. For the cognitive satellite–terrestrial networks, effective resource allocation, which is a key enabling technique to alleviate the mutual interference and ensure the friendly coexistence of two networks, has also been widely studied. The authors in [20] presented several basic scenarios and system models for cognitive satellite–terrestrial networks, where satellite and terrestrial networks can operate as primary and secondary systems, respectively, or vice versa. In regard to spectrum sharing, cognitive satellite–terrestrial networks can operate on various modes, e.g., underlay, overlay, and interweave [21]. The underlay mode, in which the cognitive system is allowed to share the spectrum licensed to the primary system without causing excessive interference at the primary user, is especially attractive due to its effective spectrum utilization. In this context, various resource allocation schemes were studied to optimize the performance of cognitive satellite–terrestrial networks. In particular, the authors in [22] designed a cognitive zone to guarantee the primary satellite communication while providing high service availability to secondary terrestrial users. The authors in [23] proposed a joint carrier–power-bandwidth allocation scheme to maximize the throughput of the satellite network, which operates in the microwave frequency band. In [24], the authors introduced a power allocation scheme that can optimize the effective capacity of terrestrial communications for given quality of service (QoS) requirements while guaranteeing the outage probability (OP) of satellite communications. In [25], the authors conducted power allocation to maximize the achievable rate for cognitive hybrid satellite–terrestrial networks with amplify-and-forward (AF) relays. In addition, for real-time satellite applications in cognitive satellite–terrestrial networks, the authors in [26] conducted power control to maximize the delay-limited capacity without degrading the communication quality of the primary terrestrial user.

From the above analyses, we can see that there are already some works devoted to improving the spectrum utilization from the perspective of full frequency reuse patterns and cognitive radio, along with effective co-channel interference (CCI) mitigation schemes. However, relying on either of these two technologies alone is not enough to meet the capacity demands of satellite communication systems in 5G and beyond, which motivates the work in this paper. The novelties and contributions of this paper are surmised as follows.

- To further enhance the capacity of satellite communication systems, we firstly propose a CR-based multi-beam HTS communication architecture in this paper, where the satellite network shares the spectrum with the terrestrial network and operates in an FFR pattern. We also analyze the characteristics of this system from the perspective of large-scale antennas and multi-gateways.
- We then review the challenges and design requirements of the RRM in the cognitive HTS system, which paves the way to the design of resource management. In particular, in this multi-beam satellite communication system with large-scale users, CCI comprised of inter-system CCI and intra-system CCI is the major obstacle for increasing the overall system throughput [28]. As a result, the RRM in such a complex interference scenario is a challenging problem, which is the focus of this paper.
- Based on the challenges and the design requirements of RRM, we propose a joint spatial division and multiplexing (JSDM)-based RRM framework to eliminate interference with low training and feedback overhead. Simulation results are provided to validate the effectiveness of the proposed scheme, and some future research directions are also given.

The remainder of this paper is organized as follows. The cognitive HTS system architecture with its details is presented in Section 2, followed by the description of design requirements of the RRM in Section 3. Section 4 introduces the proposed interference-aware RRM, and Section 5 presents the simulation results. Future research directions are shown in Section 6, and Section 7 concludes the paper.

## 2. Architecture for CR-Based Multi-Gateway Multi-Beam HTS Systems

**Figure 1:**
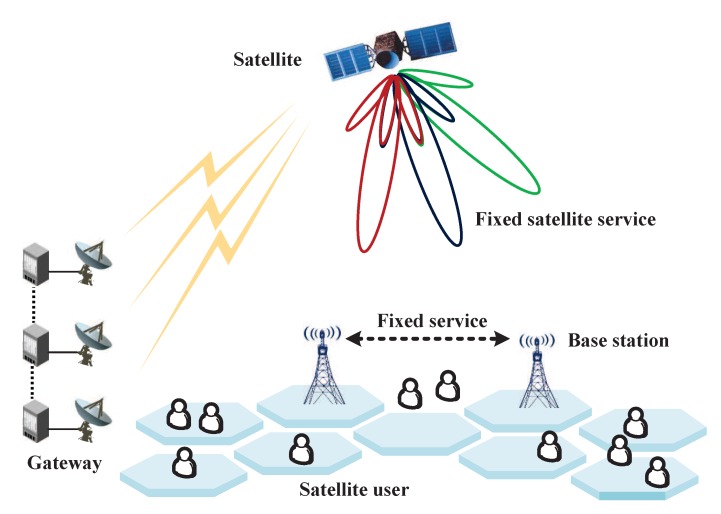
The proposed cognitive HTS system architecture.

**Figure 2:**
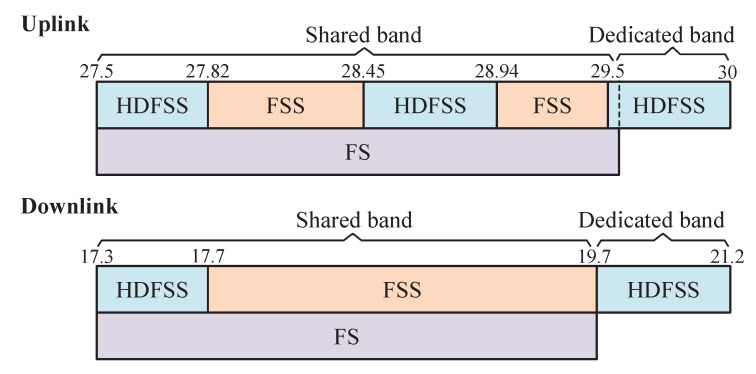
Spectrum coexistence of fixed satellite service (FSS) and high-density FSS (HDFSS) with fixed service (FS) in the Ka band.

To satisfy the ever-growing capacity requirement within the limited spectrum, we introduce a CR-based HTS communication architecture, as depicted in Figure 1, which is comprised of a geosynchronous satellite for fixed satellite service (FSS) or high-density FSS (HDFSS), multi-gateways connected with each other through fiber/wireless communication links, multiple active users, and multiple terrestrial base stations (BSs) for fixed service (FS). Here, each gateway serves a set of adjacent beams. In this way, the overall data traffic for terrestrial users can be transmitted independently by geographically separated gateways to the satellite through the feeder links [29]. Regarding spectrum sharing, a full frequency reuse pattern is employed among beams. Moreover, as shown in Figure 2, except for the dedicated 500 MHz (19.7–20.2 GHz) in the downlink and 500 MHz (29.5–30 GHz) in the uplink, HTS shares the rest of the frequency bands used for FSS in the downlink (17.3–19.7 GHz) and uplink (27.5–29.5 GHz) with other radio services, such as the terrestrial FS, small cells with millimeter waves (mmWaves), and terrestrial backhauling links.

The main characteristics of this cognitive HTS system architecture are summarized as follows.

### 2.1. Large-Scale Antennas and Spatial Correlations

In order to cope with higher data traffic demands, satellite system designers are looking for advanced satellite communication architectures. In this context, the use of large-scale antennas has recently received a lot of attention, in order to boost the system throughput by simultaneously serving multiple users. However, as the number of antennas at the satellite increases, the downlink training resources required to collect channel state information (CSI) for RRM may become prohibitively large. In this regard, the spatial correlations of satellite systems should be exploited.

Generally, spatial correlations indicate the long-term preferential directions of user channels, depending on the geometry of local scatterers and spacing of transmitting antennas. Owing to the high altitudes of satellites, channels between the satellite and terrestrial users occur along clusters of scatterers that are distributed on a narrow angular spread [30]. In this case, channels between the satellite array and a given user are spatially correlated. Moreover, since user distribution is relatively intensive from the perspective of the satellite, there may exist spatial correlations across multiple users in the considered multiuser environment. In this context, the JSDM technique can be adopted in the HTS system by organizing satellite beams in clusters, each serving multiple users experiencing similar channel statistics.

To summarize, spatial correlation provides an opportunity for CSI relaxation and becomes a promising perspective for optimizing radio resources in HTS systems.

### 2.2. Multi-Gateway

As a matter of fact, the feeder link is one of the system’s bottlenecks in the case of high-throughput multi-beam satellite systems, since it carries the entire aggregated traffic of all users between the satellite and the ground core network. Recently, some techniques have been proposed in order to optimize the feeder link spectrum resource. One solution is to move feeder links into the Q/V bands (40/50 GHz), but this makes the system more vulnerable to rain attenuation. In this regard, multiple gateways that exploit the spatial diversity and reuse the feeder link resource to distribute the communication capacity can be the preferred solution [31,32]. The advantages of employing multi-gateways can be summarized as follows: (1) Having multiple gateways means that each gateway only handles a smaller number of beams, which implies a lower signal processing complexity, and (2) in case one of the gateways fails, the traffic can be rerouted through other gateways to avoid service outages.

## 3. Challenges of RRM in Cognitive HTS Systems

In the cognitive HTS system with diverse interference, RRM plays a decisive role in system throughput promotion. From a holistic viewpoint, we summarize the challenges for the implementation of RRM in cognitive HTS systems as follows.

### 3.1. Hybrid CCI

In the cognitive HTS systems with FFR, CCI is the major obstacle for increasing the overall system throughput. Specifically, the CCI is comprised of the intra-system CCI from frequency reuse among multiple beams and inter-system CCI from spectrum sharing between HTS and terrestrial systems—referred to as hybrid CCI. To facilitate effective elimination of interference, the first issue is to establish a theoretical model to analyze the hybrid CCI, as in our prior work [33]. It is then important to study how to realize the friendly coexistence between satellite and terrestrial networks. Motivated by the promising vision of future satellite systems in 5G and beyond, the intuitive objective of RRM is to maximize the throughput of satellite communications in a multiuser scenario with diverse service requirements, while guaranteeing the normal operation of the primary licensed systems.

### 3.2. Robustness to CSI

When conducting RRM in the cognitive HTS system, a critical issue is the acquisition of the CSI at the satellite/gateway. However, the availability of perfect CSI is a major challenge in practical implementation. Remarkably, the round-trip delay from a geostationary satellite corresponds to about 500 ms. Due to the long round-trip time and time-varying channel conditions in satellite communications, any degradation of CSI (quantization errors, outdated information, or transmission errors) would compromise the performance of interference mitigation, and thus deteriorate the performance of cognitive HTS systems. Consequently, it makes much sense to design a robust RRM scheme that is insensitive to channel estimation impairments.

### 3.3. Cost Efficiency

With the tremendous demands on data traffic, there is a tendency to move the feeder link from the Ka band to higher frequency bands. Although there are larger available bandwidths as the frequency increases, the inherent hardware constraints of transceivers operating in high frequency bands make it impractical to build a complete radio frequency (RF) chain for each antenna element. This motivated the hybrid digital/analog (HDA) beamforming structure, proposed in mmWave communications, where a reduced-dimensional baseband digital beamforming is concatenated with a phase shifter network to reduce the necessary number of RF chains. In this regard, the RRM framework should be able to support cost efficient HDA precoding, in order to be valid for satellite systems spanning a large range of frequency bands.

### 3.4. Tradeoff Between Performance and System Overhead

When conducting RRM in the considered cognitive HTS systems, system information overhead mainly consists of two aspects. On the one hand, for the frequency division duplexing (FDD)-based multi-beam satellite system, downlink training and feedback overhead scales linearly with the large number of satellite antennas and will overwhelm the precious downlink resources. Thus, it is imperative to design an interference-aware RRM framework based on limited feedback CSI. On the other hand, considering the fact that the capability of on-board processing is limited for satellites, signal processing should be partially conducted at on-ground gateways. However, in the considered multi-gateway multi-beam satellite network, each gateway can only access a certain set of feeds and, consequently, the channel information of the users within its own beams. Although this kind of per-group processing is practically favorable for requiring less cost in downlink training and CSI feedback than joint group processing, it may suffer significant performance degradation due to intergroup interference. In the existing literature, there are various kinds of multi-gateway cooperation in multi-beam satellite systems, such as joint multi-gateway processing with partial CSI and partial data sharing. The more information multiple gateways exchange, the more resources are consumed, and the higher complexity of the system will be, while achieving a superior RRM solution in return.

As a result, it is interesting to investigate how to get a balanced tradeoff between the performance and the system overhead.

## 4. Interference-Aware RRM Strategy

In the considered cognitive HTS system, there exist various kinds of interference in the forward link of satellite communications. Firstly, FFR among beams would inevitably cause intra-system interference, which severely degrades the performance of satellite communications so that interference mitigation techniques are mandatory. In this context, an efficient resource management scheme is crucial to managing intra-beam interference without compromising the complexity of single-antenna receivers. Moreover, although frequency reuse between satellite and terrestrial communications can accommodate more users and services within the limited spectrum, it is always accompanied by inter-system interference at primary terrestrial receivers. Thus, when designing the resource management scheme, it is essential to take the protection of the primary terrestrial system into account.

Regarding the aforementioned design challenges of RRM for cognitive HTS systems, we first propose a JSDM-based RRM framework for the cognitive HTS system in this section. Based on the proposed framework, user grouping and two-stage precoding are studied, in order to combat the performance degradation caused by interference.

### 4.1. JSDM-Based RRM Framework

By exploiting the aforementioned characteristics of spatial correlations in HTS systems with large-scale antennas, JSDM is an attractive means of reducing the channel training and the CSI feedback overhead while maximizing the throughput in multibeam HTS systems. The main idea of JSDM lies in partitioning users into groups with similar channel characteristics and employing a two-stage precoding scheme [34]. Different groups are sufficiently separated in the angle domain. Then, the structure of the channel covariance matrix can be used to reduce the dimension of the effective channel, resulting in reduced dimension channel training and low CSI feedback overhead. The two-stage downlink multiuser precoder can be obtained by concatenating a pre-precoding matrix that depends only on the channel’s second-order statistics with a multiuser precoding matrix that depends on instantaneous realization of the resulting reduced dimensional effective channel. The pre-precoding matrix is utilized to minimize the intergroup interference, while the multiuser precoding matrix is employed to minimize the intragroup interference.

**Figure 3:**
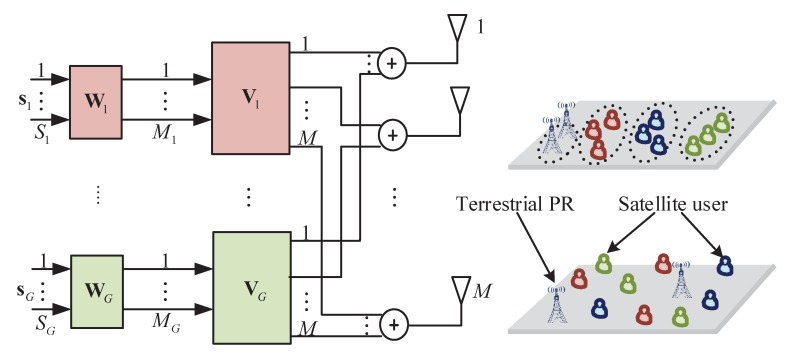
Framework of the joint spatial division and multiplexing (JSDM)-based radio resource management (RRM) in cognitive HTS systems.

In particular, since the transmitting directions of terrestrial and satellite user terminals are different, different terminals should be divided into different groups. Thus, *K* satellite users are partitioned into *G* groups with similar channel characteristics, and terrestrial primary receivers (PRs) are categorized into an individual group, as shown in Figure 3. These groups are served with a two-stage downlink precoding scheme. In order to protect the terrestrial PRs from inter-system interference, the satellite needs to obtain the channel state information of satellite–PR links when conducting resource allocations. Specifically, the first-stage precoder $V_{g}\in C^{M\times M_{g}}\left(g=1,\cdots ,G\right)$ is designed at the satellite, where *M* is the number of antennas at the satellite and $M_{g}$ is the number of beams corresponding to the *g*-th gateway. The second-stage precoder $W_{g}\in C^{M_{g}\times S_{g}}\left(g=1,\cdots ,G\right)$ is designed at the *g*-th gateway, where $S_{g}$ is the number of users scheduled by the *g*-th gateway.

In this paper, $V_{g}$ is generated through an approximated block diagonalization (BD) algorithm to eliminate inter-group interference semi-statically based on long-term CSI, i.e., second-order statistics of the channel. For approximated BD algorithm, the precoding matrix of one user must be in the null space of the others, which is determined through a threshold α. This threshold plays a decisive role in controlling intergroup interference and allowing frequency reusing among different systems. The role of the pre-precoding is to reduce the dimensionality of the effective channel by exploiting the near-orthogonality of the eigenspaces of the channel covariance matrices of different user groups. Next, with reduced dimensions on the effective channel, the second-stage dynamic precoder $W_{g}$ can be designed based on minimum mean square error (MMSE) or zero-forcing (ZF) algorithms to distinguish users within a group by suppressing intra-group interference. Through the designed two-stage precoding, both the intra-system CCI and inter-system CCI can be effectively managed.

As it is able to significantly reduce the overhead in both downlink training and uplink CSI feedback, JSDM enables practical applications of large antenna arrays in satellite communications, even operating in the FDD mode, where uplink/downlink channel reciprocity cannot be exploited. In addition, JSDM can be applied to an HDA implementation, where the pre-beamforming stage is implemented in the analog RF domain and the second precoding stage is implemented in the baseband. Hence, the proposed JSDM-based RRM framework is generic and can be adaptive to various communication systems.

### 4.2. User Grouping

In the JSDM-based RRM framework, user grouping plays a decisive role in the efficiency of the interference cancellation [35,36]. Aiming at eliminating both the intra-system CCI among satellite users as well as inter-system CCI between satellite and terrestrial users to protect primary terrestrial communications, we divide satellite users and terrestrial users together into several groups. Specifically, in order to suppress the inter-group interference, the pre-beamforming matrix $V_{g}$ for the *g*-th group shall be carefully designed based on all group centers $R_{1},\cdots ,\mathrm{and}\;R_{G}$. Moreover, since only the users within the group can be scheduled for each group, user grouping also has significant impacts on subsequent user scheduling.

When partitioning users into groups, the following qualitative principles need to be obeyed. Firstly, users in the same group have channel covariance eigenspaces spanning approximately a given common subspace, which characterizes the group. Secondly, the subspaces of groups are mutually orthogonal, or at least have empty intersections. According to these two principles, users can then be grouped based on a certain similarity metric. However, it is usually assumed that each group managed by a single gateway comprises geographically close users [37]. In reality, users do not come naturally partitioned in groups with similar spatial correlations. Thus, user grouping based on geographical locations may degrade the system performance dramatically.

To efficiently exploit the superiority of the proposed JSDM framework, an elaborated user grouping scheme is worth being studied. With the rise of artificial intelligence, machine-learning-based clustering algorithms are attracting increasing attention from both academia and industry. In this paper, without the loss of generality, we take the K-means method [38], a heuristic algorithm in clustering, as an example to illustrate the advantage of user grouping schemes based on channel correlations. In the K-means clustering algorithm, chordal distance is used as the similarity measure which is defined as

(1) $$d_{c}\left(U_{k},B_{g}\right)={∥U_{k}U_{k}^{H}-B_{g}B_{g}^{H}∥}_{F}^{2}.$$

Here, $U_{k}$ and $B_{g}$ are the matrices of the eigenvectors corresponding to non-zero eigenvalues of user $k^{\prime }s$ covariance matrix $R_{k}$ and group center $R_{g}$, respectively.

User grouping is then formed via an iterative process. In each iteration, each user is assigned to the group with the minimum chordal distance. Then, the group center is updated using a unitary matrix of users currently associated with the group as

(2) $$V_{g}=\Upsilon \left\{\frac{1}{\left|K_{g}\right|}\sum_{k\in K_{g}}U_{k}U_{k}^{H}\right\},$$

where $K_{g}$ denotes the user set of group *g* and $\left|K_{g}\right|$ denotes the size of group *g*.

In this way, user grouping can sufficiently utilize the spatial correlation to reduce inter-group interference and lay a good foundation for the subsequent two-stage precoding mentioned above.

## 5. Performance Evaluation

We conducted Monte Carlo simulations to validate the proposed resource management framework and to evaluate the system performance of the introduced cognitive HTS system. It was assumed that the bandwidths of satellite and terrestrial system were 0.5 and 2 GHz, respectively. The multi-beam satellite was assumed to be equipped with a uniform rectangular array with $N_{t}$ antennas, and the terrestrial BS was equipped with a single antenna. Within the satellite coverage with an area of 40,000 $km^{2}$, terrestrial BSs and *K* users were uniformly distributed, where there were β BSs per 10,000 $km^{2}$. A total of $N_{g}$ gateways were considered; each gateway served one group. The total transmit power of the satellite was 60 dBW. We used the Ricean channel model to fit the forward link containing the line-of-sight (LoS) component, with free space path-loss of 190 dB. Antenna gain at the satellite was 66.5 dBi, while user terminal antenna GGTT was assumed as 16.9 dB/K in clear sky [39]. The received signal-to-noise ratios (SNRs) of terrestrial links ranged from 15 to 25 dB.

In the simulations, we first calculated the channel covariance matrices between satellite and users, which were taken as the parameter of the K-means algorithm to conduct user grouping. After user grouping, by regarding all the terrestrial BSs as a new group, we employed the approximated BD algorithm to design the first stage precoder to reduce intergroup interference with threshold α=0.8. Then, the greedy algorithm was utilized to schedule users in order to maximize the throughput of the network. Last, we employed the regularized ZF algorithm to design the second stage precoder to eliminate the intragroup interference.

**Figure 4:**
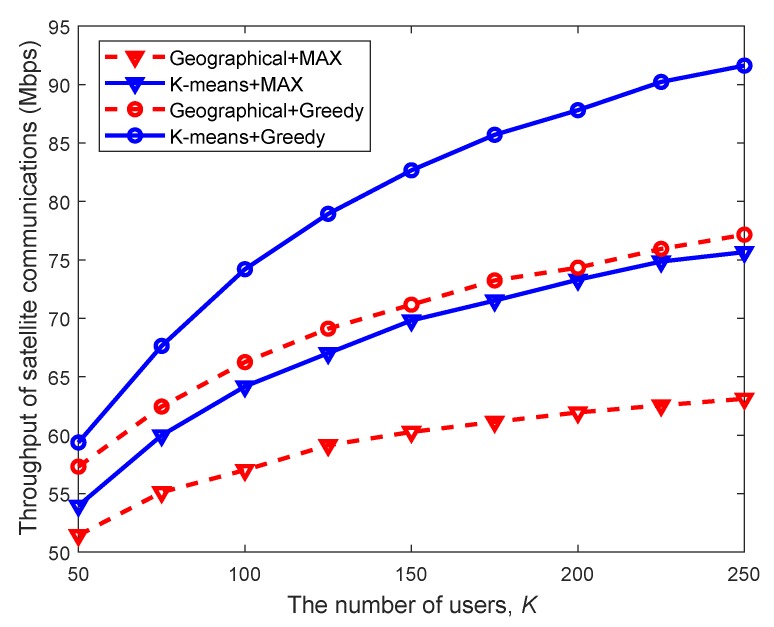
Satellite throughput versus the number of users with different schemes (under the full frequency reuse (FFR) pattern without cognitive radio (CR), $N_{t}=128$, $N_{g}=10$, and β=10).

In Figure 4, we firstly assess the performance of the proposed resource management scheme for satellite communications, where the K-means user grouping based on spatial correlations (referred to as SCUG) is compared with the existing geographical user grouping (referred to as GUG) method, combined with two classical user scheduling algorithms. One is the MAX algorithm, the key idea of which is to schedule users with the maximal signal-to-interference plus noise ratio (SINR). The other one is the GREEDY algorithm, which dynamically schedules users with potentially maximum enhancement to the system throughput among all the unscheduled users.

From Figure 4, we can observe that as the number of users increases, the satellite throughput increases linearly at the beginning and tends to remain unchanged. This is because when user size grows, the resource becomes crowded and the system performance eventually gets saturated. Moreover, the SCUG method outperforms the existing GUG method except for the case where users are sparsely distributed, verifying the benefits of exploiting spatial correlations in user grouping. Furthermore, for a given user grouping scheme, the satellite throughput obtained with the GREEDY algorithm is superior to that with MAX, which coincides with the design principles of algorithms.

**Figure 5:**
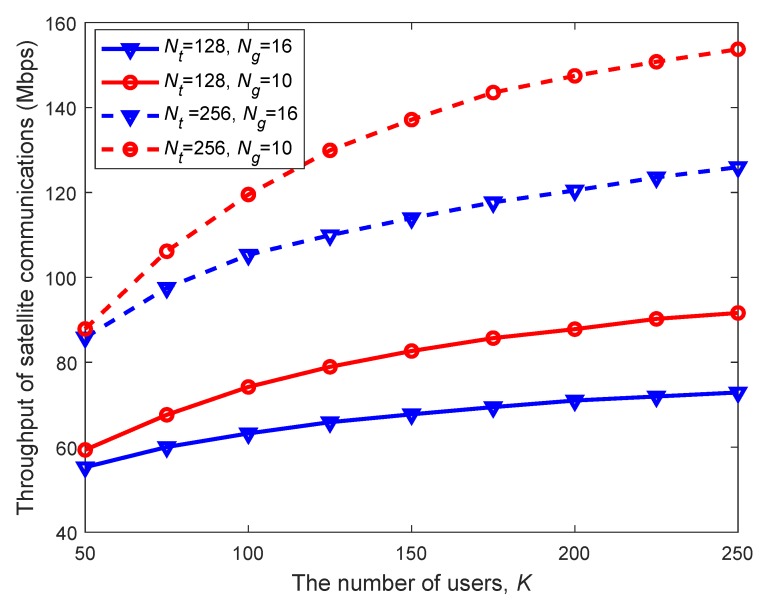
Satellite throughput with different numbers of antennas and gateways (under FFR pattern without CR, β=10).

Figure 5 illustrates the throughput of terrestrial and satellite systems with different numbers of satellite antennas and gateways in the case of FFR without CR. In general, the larger the number of antennas at the satellite is, the more throughput can be obtained. With the same number of antennas, the throughput of satellite increases as the number of gateways decreases. This is because when $N_{g}$ is small, the residual intergroup interference resulting from the approximated BD algorithm in the first stage precoder is small, and the intragroup interference can be effectively eliminated through the second stage precoder.

**Figure 6:**
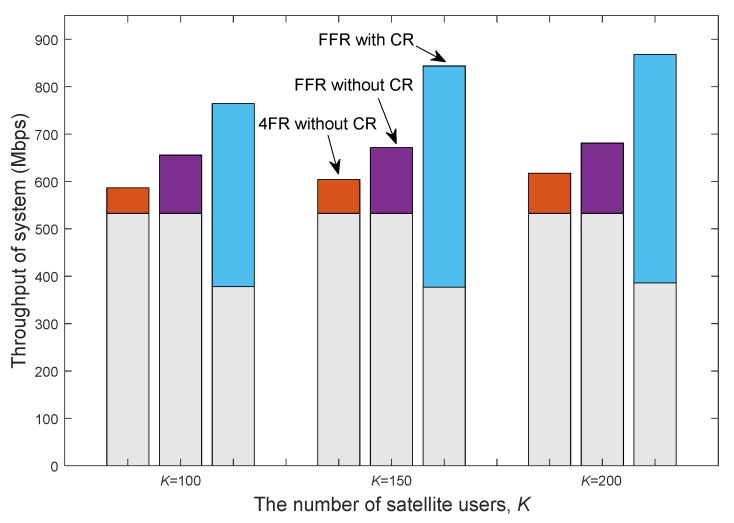
System throughput of different schemes with varying satellite user numbers. The lower and upper parts of each bar correspond to the throughputs of terrestrial and satellite communications, respectively. ($N_{t}=256$, $N_{g}=10$, and β=10).

In Figure 6, we compare the overall system throughput of the proposed cognitive HTS system architecture, referred to as FFR with CR, with those of two reference architectures: (1) Conventional 4FR configurations in satellite communications without CR with terrestrial communications, referred to as 4FR without CR, and (2) FFR employed in satellite communications without CR with terrestrial communications, referred to as FFR without CR. Since the interference between satellite and terrestrial systems varies in different schemes, the throughput of terrestrial system also varies in different schemes. Moreover, for a fair comparison, we use the same clustering method SCUG and user scheduling method GREEDY. For a given user size, the throughput gaps of different schemes demonstrate that the overall system throughput increases dramatically by incorporating the FFR pattern and CR into satellite communications. For example, when *K* = 150, we can achieve approximately 11.2% gain in the overall system throughput by employing FFR, and 39.7% gain in the overall system throughput by employing CR, which results in approximately 27% loss in the throughput of terrestrial networks.

**Figure 7:**
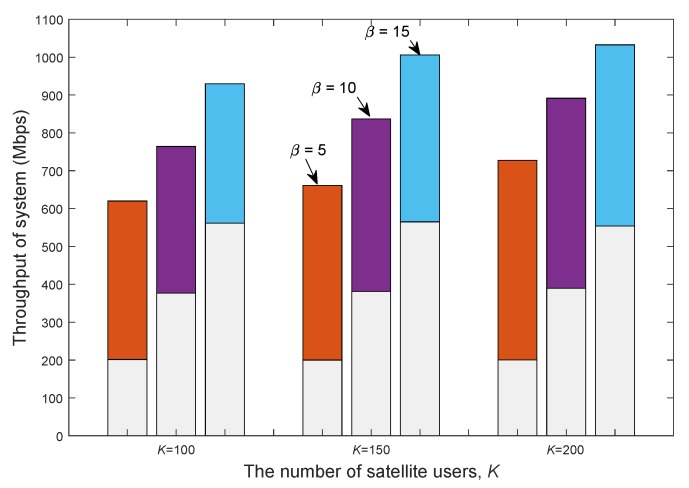
Throughput of different systems with different numbers of terrestrial base stations (BSs) versus satellite user numbers. The lower part and upper part of each bar correspond to the throughputs of terrestrial and satellite communications, respectively (under the FFR pattern with CR, $N_{t}=256$ and $N_{g}=10$).

Figure 7 presents the impacts of BS density on the throughputs of both satellite and terrestrial systems with different numbers of users. As the number of satellite users *K* increases, the throughput of the satellite system increases, while the throughput of terrestrial system remains constant. This phenomenon can be explained by two aspects. First, the transmit power of satellite remains the same. Second, the number of user groups in the approximated BD algorithm is constant and thus, the interference at the terrestrial BSs is effectively controlled through the first precoder and remains unchanged. In addition, for a given user size, as the number of BSs β increases, the throughput of the terrestrial system increases, while the throughput of the satellite system decreases. This is because more BSs results in severer interference at the satellite users.

## 6. Future Research Directions

### 6.1. SDN/NFV-Enabled HTS Systems

Leveraging the concepts of software and virtualization, software-defined networks (SDN) and network function visualization (NFV) are expected to be attractive paradigms for satellite communications. The introduction of SDN and NFV paradigms into the current evolution trend of HTS could improve key system characteristics such as flexibility, customization, scalability, etc. However, there are several technical challenges which remain to be solved when turning the SDN-/NFV-enabled HTS systems to practical implementation. Firstly, an efficient and cost-effective deployment strategy for SDN controllers and SDN-enabled smart gateway diversity architecture should be carefully designed. Moreover, automatic and programmable handover operation between gateways is also the cornerstone of SDN-enabled HTS systems. In addition, orchestrating the network resources through network slicing according to the network QoS indicators is another main challenge.

### 6.2. Adaptive Spectrum-Sharing Scheme

In cognitive satellite–terrestrial networks, it is important to effectively use the underutilized spectrum resources without deteriorating the primary communication. Although the considered underlay mode could improve the spectrum utilization, the accompanied CCI is the main concern, but not for overlay mode, where secondary satellite communications have minimal collisions with terrestrial communications by continuously sensing spectrum holes. It is attractive to maximize the long-term throughput of the secondary user with minimal interference to primary users and low sensing overhead. To achieve this goal, an adaptive spectrum-sharing scheme should be developed, which enables the satellite communication to switch between overlay and underlay modes flexibly.

### 6.3. Frame-Based Precoding

Nowadays, satellite communication standards embed more than one user in each frame in order to increase the channel coding gain, exploiting the super-framing structure of the latest physical layer evolutions in satellite communications. Due to the framing constraints, the same precoder needs to apply to different data of multiple receivers, which entails a modification of the conventional precoding scheme and leads to the consideration of precoding on a frame-by-frame basis. Specifically, major concerns are how to select the users that are grouped in the same frame as well as how to calculate the precoding matrix. To further improve the system performance, it is imperative to design an advanced frame-based precoding scheme while maintaining a low computational complexity.

## 7. Conclusions

In this paper, we have proposed a cognitive HTS system architecture to achieve spectrally efficient and high-throughput broadband satellite communications. The practical challenges of designing resource management in cognitive HTS systems have also been analyzed. By exploiting the spatial correlation of satellite channels, we have provided an interference-aware RRM scheme based on JSDM, which can effectively manage the hybrid interference with a reduced training and feedback overhead. Simulation results have shown the effectiveness of the proposed resource management framework and demonstrated the viability of the cognitive HTS network architecture. Finally, future research challenges have also been discussed.

## Figures and Tables

**Table 1:** The main reasons and the related solutions for limited throughput of high-throughput satellite (HTS) systems.

Problems	Reasons	Solutions	References
Limited throughput of HTS systems with underutilized spectrum	Partial frequency reuse pattern [8,9,10,11,12]	Full frequency reuse pattern with interference manag ement [13]	[14]: Multiuser interference detection and cancelation on the user side
			[15,16]: Advanced precoding schemes at the transmitter side
	Exclusive regulation of spectrum assignment [17]	Cognitive radio-based spectrum sharing between HTS and terrestrial systems with interference management [18,19]	[20,21]: Various interference scenarios and cognitive modes in cognitive satellite terrestrial networks
			[22]: Cognitive zone to guarantee the primary communications
			[23]: A joint carrier–power- bandwidth allocation scheme
			[24,25,26]: Power allocation with interference-power constraints
